# Making the most of scarce biological resources in the desert: Loptuq material culture in Eastern Turkestan around 1900

**DOI:** 10.1186/s13002-024-00660-5

**Published:** 2024-02-26

**Authors:** Patrick Hällzon, Zulhayat Ötkür, Sabira Ståhlberg, Ingvar Svanberg

**Affiliations:** 1 Department of Linguistics and Philology, Uppsala University, Uppsala, Sweden; 2 Institut Für Slavistik, Turkologie Und Zirkumbaltische Studien, Johannes Gutenberg University of Mainz, Mainz, Germany; 3 Department of Cultural Anthropology and Ethnology, Uppsala University, Uppsala, Sweden; 4 Institute for Russian and Eurasian Studies, Uppsala University, Uppsala, Sweden

**Keywords:** Cultural ecological services, Ethnobiology, Fisher-foragers, Food-getting technology, Historical research, Homecraft, Local knowledge, Marsh habitat, Methods of transport, Turkology

## Abstract

**Background:**

Most fisher-gatherer communities we know of utilized a limited number of natural resources for their livelihood. The Turkic-speaking Loptuq (exonym *Loplik, Loplyk*) in the Lower Tarim River basin, Taklamakan desert, Eastern Turkestan (Xinjiang), were no exception. Their habitat, the Lop Nor marsh and lake area, was surrounded by desert and very poor in plant species; the Loptuq had to make the most of a handful of available biological resources for housing, furniture, clothing and fabric, fishnets and traps, tools and other equipment. The taxa used by the Loptuq were documented by foreign explorers at the end of the nineteenth and beginning of the twentieth centuries, prior to the forced resettlement of the group in the 1950s and subsequent destruction of their language, lifestyle and culture.

**Methods and sources:**

Ethnobiology explores the relationship between humans and their environment, including the use of biological resources for different purposes. In several aspects, historical ethnobiology is more challenging; it studies this relationship in the past and therefore cannot verify results with informants. As the present study discusses an extinct culture on the basis of literary and material sources, we apply a method called *source pluralism*. This approach allows the inclusion and combination of a wide range of data and materials, even scraps of information from various sources, with the aim to understand phenomena which are sparsely mentioned in historical records.

Travel reports by Swedish, British, German, American and Russian explorers together with linguistic data provide the most important sources for understanding Loptuq interaction with the environment and its biota. Especially the large number of toponyms and phytonyms recorded by the Swedish explorer Sven Hedin and materials from his expeditions, including voucher specimens kept in Stockholm in the herbarium of the Swedish Natural History Museum, and objects of material culture in the collections of the Ethnographical Museum, are crucial for our analysis about local knowledge among the Loptuq. Illustrations and photographs provide us with additional information.

**Results:**

The question of how the Loptuq managed to survive at the fringe of a desert, a marsh and a lake which changed its location, intrigued all foreign visitors to the Lop Nor. The Loptuq’s main livelihood was fishing, hunting and gathering, and their material culture provided by plants and other organic materials included their usage, consumption and trade. Only a handful of species formed the basis of the Loptuq material culture, but they had learned to use these specific plants for a variety of purposes. The most important of these were Lop hemp, *Poacynum pictum* (Schrenk) Baill., the riparian tree Euphrates poplar, *Populus euphratica* Olivier, and the aquatic common reed, *Phragmites australis* (Cav.) Trin. ex Steud. Several species of tamarisk were used for fuel and building fences. A few plants were also harvested for making foodstuffs such as snacks and potherbs. In addition, the Loptuq also used fur, bird skins, down, feathers, mammal bones and fish bones for their material needs. The habitat provided cultural ecological services such as motifs for their folklore, linguistic expressions and songs, and the Loptuq engaged in small-scale bartering of plant products and furs with itinerant traders, which ensured them with a supply of metal for making tools.

**Conclusion:**

This article discusses the now extinct Loptuq material culture as it existed more than a hundred years ago, and how the scarce biological resources of their desert and marsh habitat were utilized. Loptuq adaptation strategies to the environment and local knowledge, transmitted over generations, which contributed to their survival and subsistence, were closely connected with the use of biological resources.

For this study, a comprehensive approach has been adopted for the complex relationships between human, biota and landscape. The Loptuq are today largely ignored or deleted from history for political reasons and are seldom, if at all, mentioned in modern sources about the Lop Nor area. Their experience and knowledge, however, could be useful today, in a period of rapid climate change, for others living in or at the fringe of expanding deserts.

## Background

Human usage of plants includes far broader aspects than only food or medicine: the study of material culture is a core research area for ethnobiology, too [1]. Human–plant relationships generate many biocultural domains. Archaeological, ethnobiological and ethnographical research worldwide demonstrate that plants and plant materials have been utilized by humans for a wide range of purposes during millennia, such as producing textile fibres, equipment, dwellings, animal feed, colorants, cosmetics, fuel, dyes, food, hygienic products, attrahents, pesticides, poisons, remedies, repellents and stimulants. Plant-derived products have also been used for ornamental purposes and rituals. A number of plants have found important applications in the material culture of foraging, pastoral and agricultural societies [2]. The botanical knowledge, especially of hunter-fisher-gatherer groups, is usually well-developed and detailed; consequently, it is an important topic for ethnobiological research, which already pioneering studies in the field indicate [3, 4].

Yet, fisher-forager knowledge and use of botanical materials such as plants and trees remain an understudied topic [5]. This lack of knowledge regarding botanical resources becomes especially evident when discussing the use of various species for handicraft, and the production of objects for material culture such as fabrics, gear, housing, tools, boats and traps [6]. Fisher-foragers have always been dependent besides fishing also on surrounding plant resources, not only as a food supplement, but also for dwellings, equipment, implements, tools and textiles [7, 8]. Although animal products can provide clothing [9–11], tools [11] and even transport [13, 14], plant materials offer more opportunities for enriching the material culture [1]. Further, it is essential to study fisher-foragers’ knowledge about and interest in those animals and plants that have no obvious economic value. This latter aspect is rarely considered in ethnobiological research, where a general trend is to focus on economically valuable products [15, 16]. Another understudied topic relating to fisher-foragers’ food is microbiota (lactic acid bacteria, moulds, yeast, etc.). Such ingredients have been vital for food fermentation processes, but they are very complicated to study in a historical ethnobiological context [17, 18].

Fisher-forager peoples have lived not only in coastal areas, but also along lake and river shores and in and around marshes. Until recently, the Eurasian steppe, semi-deserts and deserts were inhabited by several groups of hunter-fisher-foragers. The so-called *tugay* forests, typical riparian forests along rivers and lakes in inner Eurasia, provided ecosystem services for humans for millennia [19]. The first Western descriptions of fisher-foragers in the centre of the Eurasian continent appeared in travel reports from the late nineteenth century. In 1874, the English geographer Herbert Wood (1837–1879) accompanied an expedition of the Imperial Russian Geographical Society to examine the Amu Darya River and the region around the Aral Lake. In the northern part of the river delta, he found Qaraqalpaq fishermen, who depended on reed as construction material for their dwellings, fences and other equipment [20].

If we turn eastwards to the Tarim Basin, late nineteenth-century travellers encountered the Loptuq people, also known under the exonym Loplik; modern Chinese *Luóbù* 罗布. They were mentioned already in Chinese eighteenth-century sources as a community of fishermen and gatherers [21]. During the second half of the nineteenth century, several explorers and ethnographers from Europe and America visited and documented their settlements in Eastern Turkestan (now the political unit Xinjiang Uyghur Autonomous Region, P.R. China). The first Westerners to visit the Loptuq were members of the British Yarkand Expedition to Eastern Turkestan in 1873. This expedition aimed at establishing trade with the province and was headed by the Anglo-Indian administrator and diplomat Sir Douglas Forsyth (1827–1886), who noted:

“There is no cultivation in Lob. The people live on fish, and the products of their flocks and of the chase. In April and May they collect and eat raw the soft young shoots of a water plant called *suya* [*soyo*]; it has a long stem like reed, but is different both from the *comush* [*qamïš, Phragmitis australis*] and the chígh [*čige*, *Poacynum pictum*] [22].”

In 1876, the Polish-Russian explorer Nikolay Przhevalsky (1839–1888) visited the Loptuq during his second Central Asian expedition. Przhevalsky referred to them as Qara-Qoshun, since they lived on the shores of a lake called by this name, and around the marshes and reed belts of the Lower Tarim River and the lake Lop Nor. They led a simple life, sustaining as fishermen, foragers and hunters, he noted; yet, despite extreme ecological and climatic conditions, which limited the possibilities for human life in the area, they managed to survive. With the help of a rather simple food-getting technology, they would fish, trap waterfowls and hunt wild camels, antelopes and other mammals. They also gathered wild plants for housing and transport, and fibres for making cloth and fishing nets [23].

Forsyth’s observations were confirmed by Przhevalsky. Both observed the Loptuq from a settled, agricultural-society-focused viewpoint; this bias is reflected in their consternation about the subsistence practices of a for them utterly alien fisher-forager culture. Przhevalsky visited the Loptuq again during his fourth expedition in 1885 [24], but neither of these early travellers remained for long, or were capable of or interested enough in deepening their understanding about the Loptuq fisher-gatherer lifestyle.

Twenty years after Przhevalsky’s first visit, the Swedish explorer and geographer Sven Hedin (1865–1952) stayed with the Loptuq for a few weeks in the spring of 1896 [25]. Four years later, he remained with them for over a year, and in the 1930s he again visited them, gathering ample linguistic and other materials and observations during all expeditions. Around 1900, Loptuq life had already changed through increased contact with the outside world. Hedin observed that still most of them subsisted on fish captured in nets placed in their family-owned *čap*γ*an*, narrow channels they made and kept open in the dense reed belts. They also gathered duck and waterfowl eggs and plant shoots, and produced most of their necessities from the limited botanical resources in the area. Their material culture was not very rich, but taking into account the scarce resources available in their habitat, it was of a surprising variety [26].

The period around 1900 is important to study, because during this time several changes occurred, and part of the Loptuq had started to drift away from their traditional lifestyle. Sven Hedin arrived at the last moment to document a way of life and local knowledge, which was to disappear completely within a few decades. Some Loptuq had already moved to the nearby garrison and market town Charkliq (Chinese Ruòqiāng) for economic reasons, where they lived and worked alongside so-called *Chantou* (‘Turban-heads’) or Eastern Turki, today known as Uyghur, and some Chinese. Other Loptuq had settled in the oasis town Miran, and many people from the river or lake hamlets would go to Miran in summer for trade or farm work [27].

## The Loptuq people

### Study area and ethnographic information

**Fig. 1:**
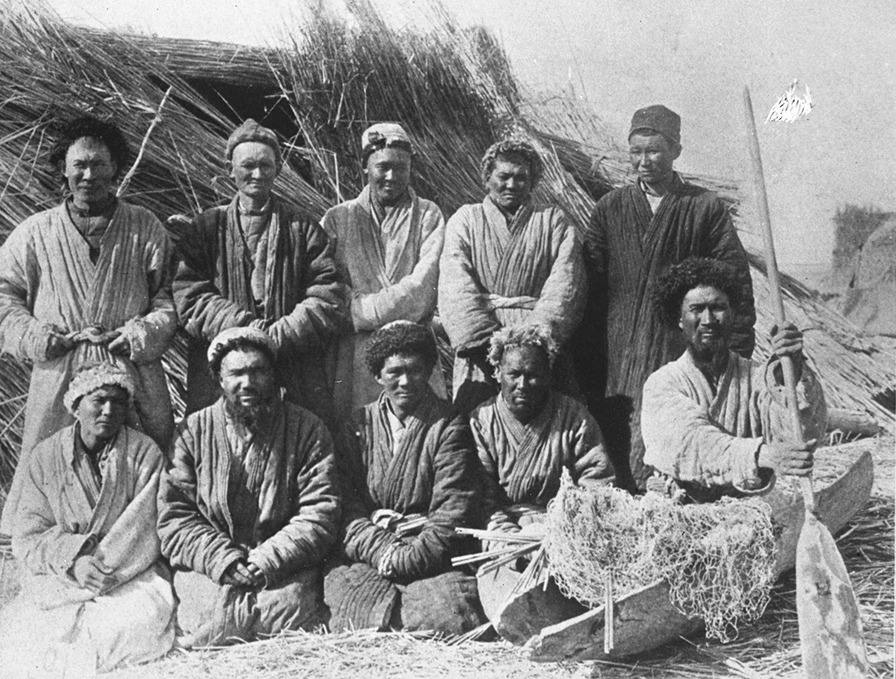
Loptuq fishermen with nets and paddle in the village Tikenlik (Photo M.A. Stein, *Ruins of Desert Cathay* 1912)

The Loptuq spoke a Turkic language and lived as fishermen and foragers by the Lop Nor (Lop Lake) and along the Lower Tarim and Konche Rivers, between the Taklamakan and Kumtagh deserts in Eastern Turkestan, in what today is the political unit Xinjiang Uyghur Autonomous Region in north-western China. The ethnonym can be translated as ‘inhabitant of Lop country.’ Until the end of the 1950s, their culture, society, economy and language differed from the surrounding Uyghur, but similarly to dozens of other groups, they have not been recognized as a separate ethnic group in P.R. China. Instead, they are, if mentioned at all, identified as Uyghur, or if referred to especially, as Loplik-Uyghur. When talking about themselves, the Loptuq used the endonym *Loptuq*; the exonym is Loplik, Loplyk or Lopluk [28] (Fig. 1).

Extensive ecological changes due to climate change during the past century, epidemics and civil wars, and since the 1950s, dam projects which have dried up the rivers, marshes and the lake Lop Nor, and nuclear tests at the Lop Nor site have contributed to destroy the habitat of the Loptuq. The lake finally dried out by 1964. Their culture and livelihood were annihilated by the Chinese authorities, who resettled them in the 1950s far away in a few oasis settlements, where they were assimilated as peasants among the majority Uyghur population [28–30].Today, having lost their language, culture and traditions, the Loptuq keep only a few memories of their folk songs [28, 31].

Most scholars agree today with a Loptuq oral tradition, documented by some travellers, that they were earlier divided into several subgroups with various origins, the earliest of whom arrived in the region probably only a few hundred years earlier: *Qara Qošulluqtar*, who had moved downstream along the Tarim River; *Yallar*, traders and Muslim missionaries from towns in Eastern Turkestan (Turpan, Qomul, Kashgar, etc.); *Qalmaqtar*, of Kalmyk (Western Mongol) origins; *Judaqtar*, also of Mongol origins, possibly from nearby regions Gansu and Amdo (Chinese Qinghai); *Qalučilar*, related to *Judaqtar*; and *Kirghizzar*, whose ancestors allegedly were Kirghiz [21, 32].

The diverse origins of the Loptuq confused most foreign travellers, who at the end of the nineteenth century already, were steeped in nationalist ideas of one language and one origin of every people. In 1889, the French explorer Gabriel Bonvalot (1853–1933) visited the Aqtarma village north of the Lop Nor area. Like the other travellers, he observed that the villagers lived in reed huts and subsisted on fishing, hunting and animal husbandry. They spoke a Turkic dialect, yet claimed to be Kalmyk Mongolians by descent [33]. A Russian army officer and explorer, Mikhail Pevtsov (1843–1902), pointed out several similar customs among the Loptuq and pastoralist nomads further north in Eurasia, which could indicate earlier connections or origins from another part of Central Asia or southern Siberia [34].

According to linguists, the Loptuq language would also point to more northern Turkic pastoralist origins [28, 35]. The language, however, had several local varieties with differences in pronunciation, lexicon and grammar. Many contained Mongolian words associated with the livelihood, including fishing and boating terminology, phytonyms and zoonyms [28]. In 1956, the prominent Turkologist Ädhäm Tenishev (1921–2004), a Tatar scholar who was sent to China to help document Turkic peoples, observed that the old lineage system and characteristics of Loptuq language and culture were already disappearing [32]; after the displacement of the whole group, they vanished.

### Demographic data

**Fig. 2:**
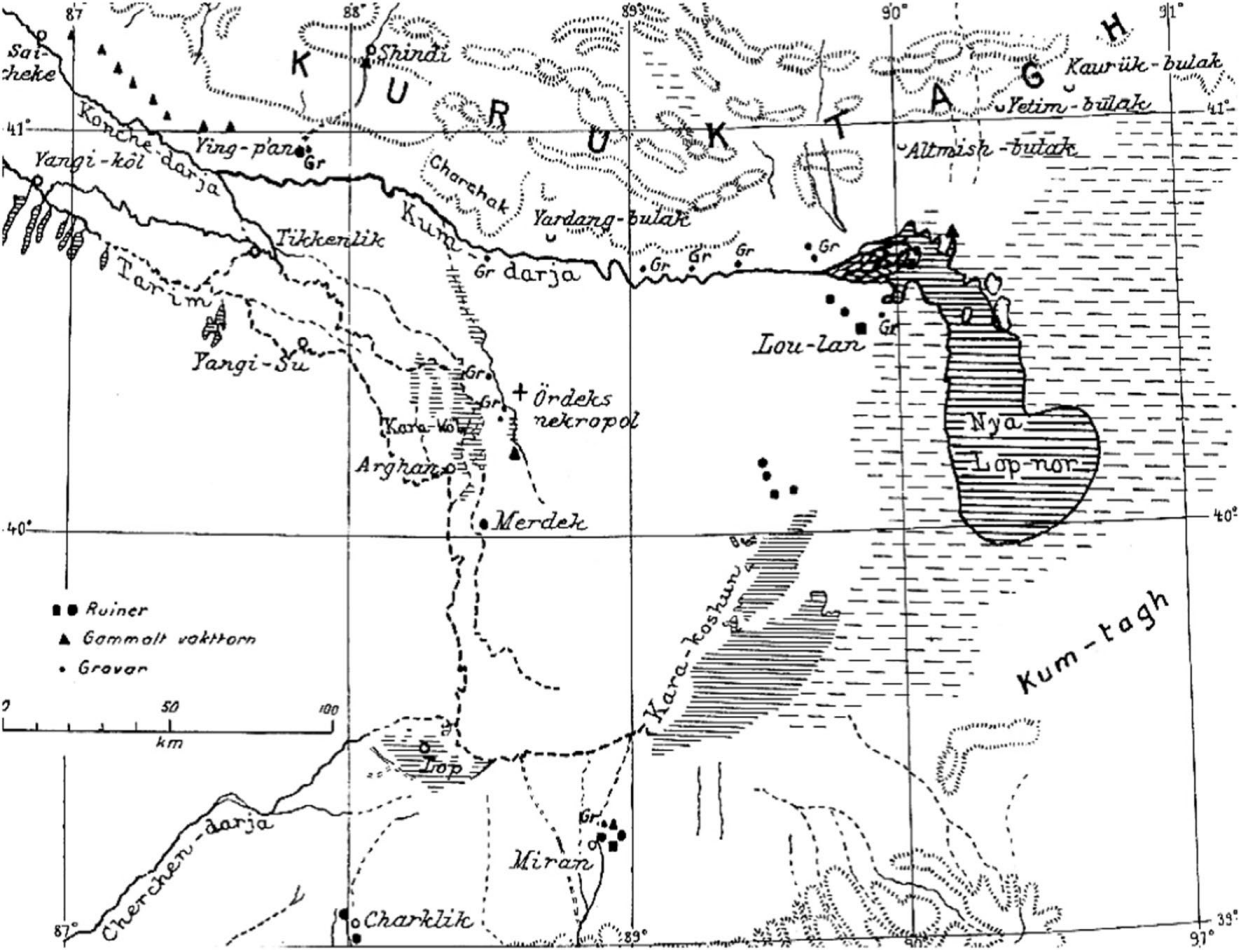
Map of the Lop Nor region by Folke Bergman, 1935. Translation of legend: Ruiner = Ruins. Gammalt vakttorn = Ancient watchtower. Gravar = Graves. Other translations: Bulak = Well. Ördeks nekropol = Ördek’s necropolis (now Xiaohe Tombs). Nya Lop-nor = New Lop Nor, the lake position and size in the 1930s during the Hedin expedition (Sven Hedin Foundation, Stockholm)

Demographic information and population estimates about the Loptuq are scarce, unreliable, and demonstrate vast variations. According to a Chinese document from the eighteenth century there were 5,000 Loptuq [21]. In the 1870s, Przhevalsky estimated their number to 70 Loptuq households or 300 individuals in eleven settlements at Lake Qara-Qoshun. Some twenty years earlier there had been as many as 550 households, but a smallpox epidemic caused a sharp decline in the population. Almost ten years later, during his second visit in spring 1885, Przhevalsky noted that the Loptuq numbered “400 souls of both sexes” [29, 36]. In 1900, however, Hedin estimated the population to be around 10,000 [37]. More than half a century later, Tenishev claimed that there were 7000–8000 Loptuq in 1956 [38]. A year later, Uyghur linguist Mirsultan Osmanov provided an almost double figure: 14,151 [39] (Fig. 2).

In the 1890s, the Loptuq main livelihood was still fishing, hunting ducks, gathering bird eggs and foraging reed shots for food, although some households had already taken up shepherding or moved to town. A limited number of individuals also devoted themselves to trade. Near the Loptuq hamlet Tikenlik there was a small Chinese administrative town called Dural, founded in 1891. Besides some eighty Chinese and a number of Loptuq, there was an *amban* (Manchu imperial official in the Qing period 1636–1912). Hedin counted around fifty houses and observed a small bazaar, where merchants from Kashgar, Khotan and Aksu in Eastern Turkestan traded [40]. Some customs among the Loptuq appeared to be new and probably should be attributed to contacts and economic changes in the last decades of the nineteenth century [35]. Around 1916, Aurel Stein found a Loptuq colony of a dozen families from the village Abdal, resettled since 1908 in the small oasis Miran [41]. In the 1930s, Hedin’s expedition counted 200 Loptuq in the oasis settlements Charkliq and Miran. After the Lower Tarim changed its course in the 1920s, many Loptuq from Abdal village moved to Miran [43]. The last figure is from 1982, when a report estimated the Loptuq population to 24,000 [43]. This has been contested by the Uyghur linguist Esmael Abdurehim, who considers it far too high [21] (Table 1).

**Table 1:** Loptuq settlements and villages around 1900

Abdal or Abdallïq	
Alqattïk-čeke	
Arγan	
Daši	
Dilpar	
Dural	
Jäkänöj	
Jäkänlik	
Jurt čapγan	
Kučuk-atam	
Lajlïq	
Qumčapγan	
Qumčeqe	
Širgečapγan	
Tikenlik	
Tiken	
Toqum	
Toquz ata	
Toquz atam	
Tusun čapγan	
Čaqliq Lop	
Čaj	
Čarχlïq	
Čegeliq-uj	

*Source*: Hedin 1900 [25]; Hällzon, Ståhlberg and Svanberg 2019 [65]

### Ecosystem and vegetation

Water was the life-blood for the Loptuq in an otherwise extreme environment and climate. The mean January temperature was –10^o^ C and the July average + 28° to + 30^o^ C. Sandstorms, *buran*, occurred regularly, and sometimes they would last for many weeks and make any foraging or fishing activities impossible. Especially during winter and spring, icy winds from the north or northwest swept the area [34, 40]. From February to April, this wind could reach a strength of ten on a ten-grade scale. Hedin noted that the Loptuq called it *qara buran* ‘black storm’, since it “carried atmospheric particles, which darkened the sky and caused dusk to appear at midday”. During other seasons, the atmosphere was comparatively calm and the winds weak and of short duration [40].

**Fig. 3:**
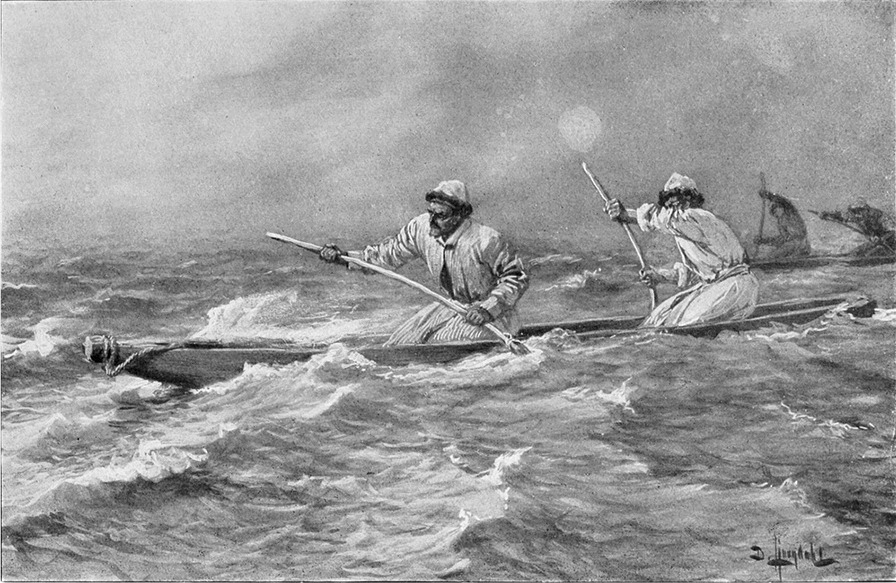
Sandstorm, *qara buran*, hits Loptuq men with their dugout canoes while out on the Lop Nor lake (Drawing by Sven Hedin)

*Qatiq buran* ‘hard storm’ and *qara buran* ‘black storm’ were the most infamous, but also the less severe *sariq buran* ‘yellow storm’ was feared. During the spring, windstorms from east and northeast were most common. The summer was especially windy, but at least part of the winter was almost calm. A serious problem was the constantly present sand and salt dust, carried around by the winds and causing chronic eye inflammation [25]. The precipitation was a mere 10 mm per year, while the evaporation was not less than 3000 mm. The Lop Nor basin was and still remains one of the most arid areas in the world [44] (Fig. 3).

### Aims, methods and sources

Although their ancestors most probably came from different locations and arrived to the desert and marsh area around Lop Nor, the inhabitants had by the end of the nineteenth century become very adept at utilizing the few available plants suitable for human consumption and the scarce material conditions in the environment. In addition to plants, their nutritional needs were mainly satisfied by different sorts of animal fat and protein (mainly fish) [35]. According to both Przhevalsky and Hedin, the Loptuq had simple needs; housing, furniture, tools and gear were very sparse. They produced most of their artefacts and goods themselves, and most of these objects were made from locally available plants and trees. Some iron tools such as cauldrons, jugs, axes, knives and fish hooks were bartered with farming neighbours in the oases, or bought from peddlers who visited the settlements. Everything else the Loptuq would produce themselves: shelter, transport vessels, clothes and tools [25, 35].

The Loptuq material culture is discussed here from a period when they were still living as fisher-foragers in the Lower Tarim River area and around lake Lop Nor. The Loptuq were heavily dependent on their surrounding environment, which provided them with many ecosystem services, both cultural and provisional [45]. This study explores the utilization of the sparse botanical and other biological resources for material needs and food. Most of the known ethnographic sources on the Loptuq have been used to identify the species utilized for various craft and nutritional purposes. The ethnographic context is important for the understanding of the folk botanical knowledge and plant use of a specific ethnic or social group; our approach is therefore ethnographic and ethnobiological, also taking into account various temporal, geographical and social dimensions [46].

The use of multiple sources is essential for ethnobiologists, and especially for historical ethnobiologists. Scattered information about historical activity contexts between humans, environment and biota can be found in various types of sources. A method called *source pluralism* has been used for this study, as it allows us to include a combination of diverse source materials, with the aim to understand phenomena that are scarcely mentioned in historical records [47]. We have found this method particularly well adapted to research in historical ethnobiology [48].

Sources with details about the Loptuq ethnography are few. In addition to Przhevalsky, who provided some interesting data, a few other travellers to the Loptuq noted details. The already mentioned Mikhail Pevtsov, who visited them in the 1880s [34], and French traveller Gabriel Bonvalot [33] conveyed a few observations of interest. In the summer of 1887, the British traveller Arthur Douglas Carey (1844–1936) made a brief visit to the Loptuq and offers some data about them, yet possibly rewritten from Przhevalsky’s travelogue [49]. A few other American, German, and French travellers also visited the Loptuq, and some of them provided observations in their published travelogues, but not much about the material culture: Thomas Douglas Forsyth (1827–1886), Ellingworth Huntington (1876–1947), Albert von Le Coq (1860–1930), Sir Aurel Stein (1862–1943), and Reginald Schomburg (1880–1958) [22, 27, 41, 50, 51].

The Swedish explorer Sven Hedin (1865–1952) was probably the visitor most devoted to the Loptuq. He spoke Turki and probably learned Loptuq during his long stay with them, and was therefore able to provide much ethnographic detail in his published and unpublished reports. Hedin made in total three visits to the Lop country and observed the area and its hydrographic conditions within a time span of almost forty years. During his first expedition to Central Asia in 1893–1897, he stayed in the area from 21 March to 23 April 1896 [40]. During his next expedition in 1899–1902, he stayed with the Loptuq for well over a year, from 26 June 1899 to November 1900 [37, 52–54]. His last expedition to Turkestan, which was carried out by car in 1927–1935, provided him with yet another chance to visit the Loptuq [55].

Hedin’s published scientific reports are, despite the fact that he was not a trained ethnographer, the most valuable sources for our knowledge about the Loptuq way of life before the changes in the mid-twentieth century. Sven Hedin was an excellent drawer and a good photographer, and his illustrations are very useful for the analysis of Loptuq material culture. Moreover, in the collection of the Museum of Ethnography in Stockholm there are fishing gear and other tools collected by Hedin and members of his expeditions, especially two Swedes, David Hummel (1893–1984) and Georg Söderbom (1904–1973), who participated in his Chinese-Swedish Expedition in 1933 (Hedin-Bendix acc. Nr 5699–5716) [56].

Linguistic materials are important sources for understanding human interaction with the environment and its biota. As K. David Harrison emphasizes, this kind of material “recognizes the mutual relationship between cultural and ecological diversity” and highlights “linguistic structures and verbal practices by which speakers conceptualize, encode, and transmit knowledge about the natural world” [57]. Important linguistic data of Loptuq was gathered by Turkologists in the early twentieth century: Russian Turkologist Sergey E. Malov (1880–1957) interviewed a few Loptuq informants in the village Charkliq in 1914 [31]; some forty years later the already mentioned Turkologist Tenishev carried out fieldwork in the same region. Later, some Uyghur scholars have conducted ethnographic, folkloric and ethnographic research among the displaced Loptuq: Esmael Abdurehim [21], Mirsultan Osmanov (1929–2017) [39], Moydin Sayit Bostan (1935–) [58] and Abdurähim Häbibulla (1965–) who did his field work as late as 1985 [59]. The Chinese linguist Fù Màojī (1911–1988) [43] has published a comprehensive study of their language, and the German geographer and Sinologist Thomas Hoppe (1949–2010), who in 2003 visited a few remaining and abandoned settlements, has given some scientific observations of interest [60, 61].

The botanical activities of the Loptuq are also reflected in the local toponyms. Sven Hedin recorded many place names for his mapping activities in the 1890s, and these records carry valuable information of Loptuq life and landscape perceptions [62]. The Swedish Turkologist Gunnar Jarring has 1997 published a huge corpus of hundreds of hydro-, limno- and toponyms recorded by Hedin. They have also been analysed for the present study [63]. Ethnobiologists are interested in how all plants in the surroundings are perceived, not just useful plants but the entire flora. Naming of plants was an important aspect of the intricate set of relationships between human and nature. A rich herbarium with many voucher specimens collected by Hedin, especially during his second expedition, is kept in the Swedish Natural History Museum in Stockholm, and they offer further insights into Loptuq plant knowledge [64]. The local plant names recorded together with the vouchers have been analysed in a recent publication [65]. The scientific names for taxa mentioned in the text follow the Global Biodiversity Information Facility (www.gbif.org).

## Results

### Local knowledge in the waterscape

In this extreme environment, detailed knowledge of the ecological and climatic circumstances was necessary for survival, and the Loptuq transmitted information to each following generation in different ways. The local knowledge learned in childhood formed a foundation to understand what they observed, heard, perceived and experienced in the landscape, and together with what they learned during their lifetime, it contributed ultimately to their ability to live and subsist in the area. The Loptuq way of life and culture was adapted to the local ecological conditions in many ways: they lived in scattered hamlets spread out along the river and lake shores, consisting of small groups of 10–20 households. Seasonal migrations between summer and winter settlements were common. Especially the abundance of mosquitoes forced many Loptuq away from the waterways in summer [35, 60] The limited biological resources and ecosystem services provided by their habitat were mainly provisional, food, raw materials, energy, folk medicine and ornamental, but they were also used in several important biocultural domains [65, 66].

**Fig. 4:**
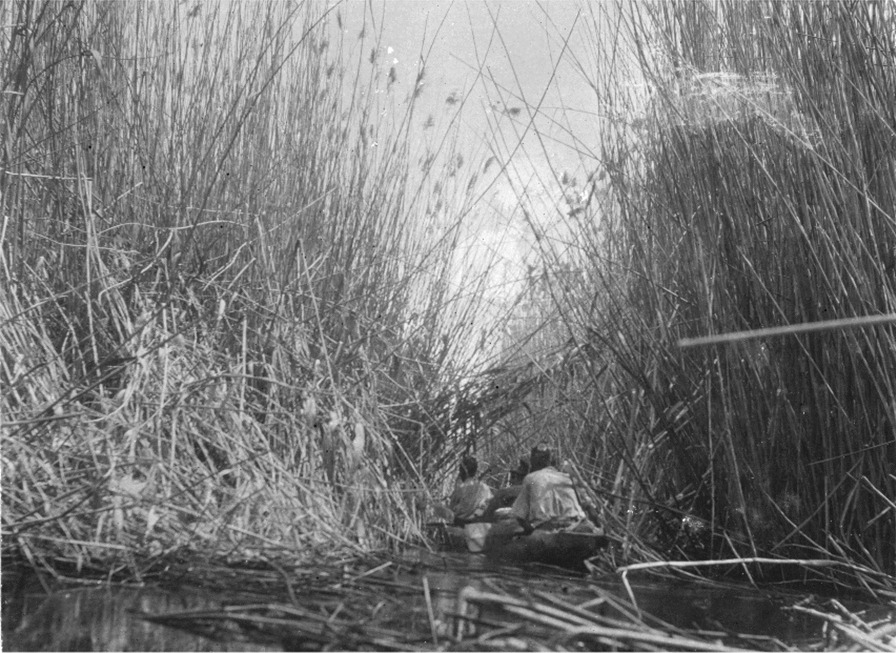
Loptuq men negotiating a man-made narrow channel, *čapγan*, in the reed belt of Lop Nor (Photo Sven Hedin)

The Loptuq were well adapted to the local conditions, but they also transformed the environment and named it while obtaining food and raw materials. Knowledge about waterways and channels formed an essential component in Loptuq perceptions of the environment and their mental geography. The most striking example is probably the reed belt in the marshlands and lake. According to Hedin, a belt of gigantic reed, “each fully 25 feet [7.6 m] in height and measuring 2–¼ inches [5–0.6 cm] in circumference at the surface of the water, stretched diagonally across the lake” where the Loptuq lived [40]. Man-made narrow channels, *čapγan* or ‘small channel’, around one metre wide, ran through the dense reeds. Some of the channels would disperse into the lake, others into further channels, and their importance for the local geography is reflected in the fact that they were named: *Qum čapγan* ‘Sandy canal’, *Jurt čapγan* ‘Village canal’, *Širge čapγan* ‘Širge’s canal’*, Tusun čapγan* ‘Tusun’s canal’*, Abdal čapγan* ‘Abdal’s canal’*, Gaday čapγan* ‘Gaday’s canal’ and so on [26, 53] (Fig. 4).

*Ilek* on the other hand indicated a wider waterway or stream, often between lake and river, a common feature among reed belts [25]. A number of place names deriving from this hydronym were documented by Hedin: *Aqilek* ‘The white river’*, Bozilek* ‘The grey river’*, Gun ilek* ‘The deep river’*, Ïdïq ilek* ‘The stinking river’*, Jaman ilek* ‘The difficult river’, etc. [26, 63]. He noted that it was important to keep the waterways open, or they would grow over. The men took care of this; often they would tie the reeds together or bend them over, so that they could move about among the walls of reed to their fishing grounds in the lake or lagoons between the reeds. Each family had their own channels and locations to put out their nets, although all had the right to use the channels for transport. Hedin observed “hundreds of nets” and “countless shoals of fish” in the clear water during a boat trip [26, 34, 40].

Naming the waterscape was an efficient strategy to describe and transmit knowledge, and also to define and decide on fishing and gathering rights of each family. The toponyms and hydronyms form a mental map closely connected with information, economic, ecological, social, etc., highly important for the Loptuq. Words especially with reed, fish, boat and waterway connotations abound in the Loptuq language, reflecting the importance of these aspects in their everyday life, culture, individual and collective memory, worldview, understanding and perception of the environment. Other dimensions appearing in toponyms are ecological features, such as plant or animal abundance in a place, outward aspect of the landscape, or economic, social or remarkable event-related naming [26]. The environment also provided cultural ecological services such as motifs for their folklore, linguistic expressions and songs [31, 33, 39, 55].

### Provision of food

**Fig. 5:**
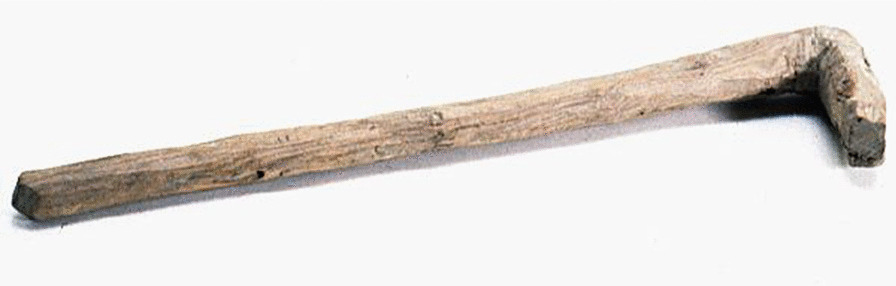
Wood mallet used to kill fish. Collected by Georg Söderbom in Charchak at Kum-darya during the Hedin expedition in May 1934 (Museum of Ethnography, Stockholm)

The main food-procuring activity of the Loptuq was fishing. This is also reflected in the abundance of terminology related to fishes and tools. The inhabitants in Lop Nur used *gölme* ‘fishing nets’, *čaŋγaq* ‘a type of hook’, *sačγaq* ‘spear’ and *qarmaq* ‘fish hook’ [31, 59, 67], and their most important possessions and tools were the above-mentioned and dugout canoes, traps and the nets made of *Poacynum* fibre. Both men and women knitted nets. The fishing season lasted from early spring to late autumn, but in times of need, fishing could be done also in other seasons. The Loptuq used different techniques for fishing, depending on the place and fish species they wanted to catch. Nets and seines were put into the lakes or rivers from canoes, and the fish were driven into the nets by men and women alike. In the spring, after the flooding of the river, fish remained in isolated lagoons and were easy to catch. Spring catches were dried unsalted in the sun for the winter and then stored in reed huts. Sven Hedin also noted that fishing was done along the Tarim River or in the lake when a sheet of floating ice, *kömül* or *qade*, formed on the surface in late autumn [23, 25, 31, 63]. According to Pevtsov, the Loptuq at Qara Qoshun (Kara Koshun on map. Figure 2.) distinguished five species of fish (Table 2) [34]. *Minlaj* is most probably a loanword from Chinese for the fish *miánlǐ.* Before 1958, the Tarim River was home to around 15 native fish species [68]. A few other fish species known by the Loptuq were mentioned by travellers, but cannot be identified; among them is Hedin’s and Malov’s *laqu*, the biggest fish in Lop Nor with a big head [31, 63, 66, 69]. Another unidentified large fish was the *juγan* [63]. More recently, the Uyghur ethnographer Häbibullah Abdurehim mentions a few other species: *bélijan, tirna, patmačuq* (over three metres and 15 kg) and *loha* (more than 25 kg) [70]*.* Fresh fish were eaten boiled, while dried fish were first steeped in salt water and then fried [24] (Fig. 5).

**Table 2:** Loptuq fish names in Lop Nor

egej balïq = Ili marinka, *Schizothorax eurystomus* Kessler, 1872	
otur balïq = Tarim schizothoracin, *Schizothorax biddulphi* Günther, 1876	
tazek balïq = Kashgarian loach, *Hedinichthys yarkandensis* (Day, 1877)	
it balïq = scaly osman, *Diptychus maculatus* Steindachner, 1866	
laqu (loqo), minlaj = bigheaded carp, *Aspiorhynchus laticeps* (Day, 1877)	

*Source*: Svanberg and Ståhlberg 2020 [26]

Plant diversity was poor in the Lop Nor area, which might explain the limited use of plants both for food and material culture among the Loptuq. Natural vegetation was sparse in the region overall and poor in the number of species. Useful species were few. For example, in 1979–1982, the Lop Nor Scientific Expedition collected in all 36 species of plants of 13 families, mainly Chenopodiaceae and Compositae and 26 genera. All these species are drought-resistant, salt-tolerant shrubs and perennial grasses. The flora and also the fauna were according to the explorers more species-rich over a hundred years earlier, when water availability was more abundant; today the area is completely arid.

**Fig. 6:**
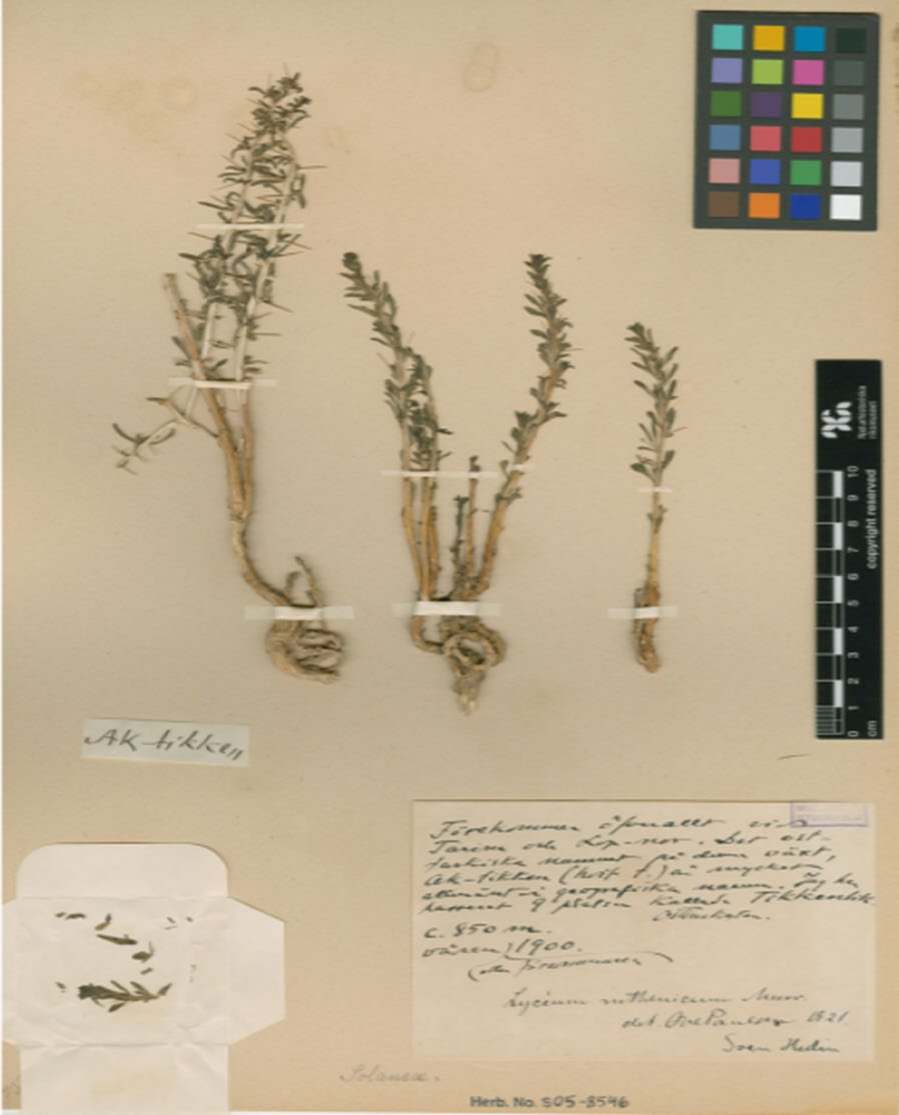
Voucher specimen of wolfberry, *Lycium ruthenicum*, gathered by Sven Hedin at Lop Nor in the spring of 1900. On the label he noted the local plant name Ak-tikken, i.e. *aq tikän,* ‘white tikän’, with a note that it is very common, and a popular place name in the Lop Nor region (Swedish Museum of Natural History, Stockholm)

According to Hedin, the Loptuq villagers of Tikenlik ‘Thistle place’ subsisted on fish, stalks and sprouts of *jäkän* or southern cattail, *Typha domingenis* Pers., which was common in the reed belts. In addition to fish they also hunted wild ducks, and took eggs from wild ducks and geese [53], gathered berries and consumed the fruits, known as *qarγa-jigdä* ‘crow berry’, of the oleaster, *Elaeagnus angustifolia* L. [63, 73, 74]. Häbibulla Abdurehim notes that in the 1930s, cattail pulp, wheat grains and oleaster fruits were consumed. The latter was especially used to season fish soup [59]. According to one of the Russian linguist Nikolay Katanov’s informants in Turpan in the 1890s, these berries were prepared as a kind of soup. Each Loptuq adult would pick an average of 10–20 bags of the berries in summer. The young spring sprouts of reed, *Phragmites australis* (Cav.) Trin. ex Strud., were also used as food. In summer, panicles were harvested to produce a tough, viscous mass which was used as sugar [23]. According to a couple of sources, roots of *Poacynum pictum* (Schrenck) Baill. (Syn. *Apocynum pictum*) were roasted and served as human food [23, 33] (Table 3). Other berries were harvested as well (Fig. 6).

**Table 3:** Food plants

Reed, *Phragmites communis* (Cav.) Trin. ex Strud.	
Southern cattail, *Typha domingensis* Pers	
Wolfberry, *Lycium barbarum* L	
Oleaster, *Elaeagnus angustifolia* L	
Lop hemp, *Poacynum pictum* (Schrenck) Baill

### Hunting and animal products

**Fig. 7:**
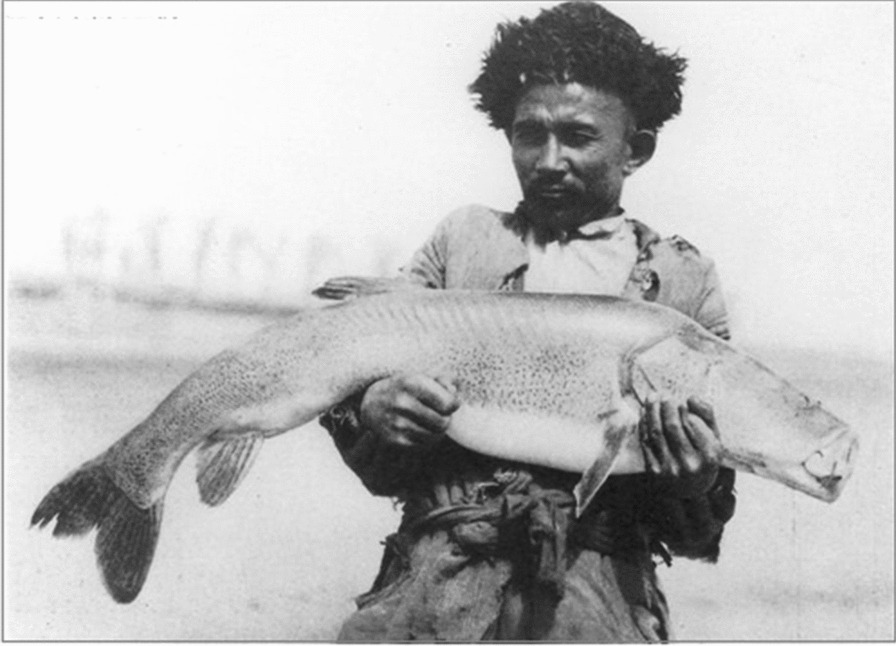
Loptuq fisherman with an impressive fish catch at the Tarim River (Photo David Hummel 1934 during the Fourth Hedin expedition)

The few fish species provided the Loptuq (Fig. 7) with food and oil, and the migratory and breeding waterfowls, cormorants, ducks, geese and swans gave them nourishment in the form of meat, fat and egg, and feathers for clothing [50], but otherwise the fauna in the Loptuq environment was poor in numbers and species. The Lop Nor Scientific Expedition collected only 127 species; 23 mammals, 91 birds, seven reptiles, one amphibian. Many wild animals, including tiger, wolf and wild hog, which were observed by explorers a century ago, have disappeared [71] (Table 4).

**Table 4:** Game and game birds

Wild yak, *Bos grunniens mutus* (Przewalski, 1883)	
Tibetan antilope, *Pantholops hodgsonii* (Abel, 1826)	
Goitered gazelle, *Gazella subgutturosa* (Güldenstaedt, 1780)	
Maral deer, *Cervus elaphus yarkandensis* Blanford, 1892	
Bactrian camel, *Camelus bactrianus ferus* Przewalski, 1878	
Asian wild ass, *Equus hemionus* Pallas, 1775	
Wild horse, *Equus ferus przewalskii* Poliakov, 1881	
Caspian tiger, *Panthera tigris virgata* (Illiger, 1815)	
Red fox, *Vulpes vulpes* (Linnaeus, 1758)	
Otter, *Lultra lutra* (Linnaeus, 1758)	
Grey-leg, *Anser anser* (Linnaeus, 1758)	
Bar-headed geese, *Anser indicus* (Latham, 1790)	
Common shelduck, *Tadorna tadorna* (Linnaeus, 1758)	
Ruddy shelduck, *Tadorna ferruginea* (Pallas, 1764)	
Pintails, *Anas acuta* Linnaeus, 1758	
Gadwall*, Mareca strepera* (Linnaeus, 1758)	
Whooper swans, *Cygnus cygnus* (Linnaeus, 1758)	
Cormorant, *Phalacrocorax carbo* (Linnaeus, 1758)	
Chukar, *Alectoris chukar* (J.E.Gray, 1830)	
Common pheasant*, Phasanius colchius* Linnaeus, 1758	
Hill pigeon, *Columba rupestris* Pallas, 1811	

*Sources*: Hedin 1903 [52]; Leche 1904 [76]; Malov 1956 [31]; Ståhlberg & Svanberg 2017 [66]

The reed waterscape contained besides fish also mussels, snakes, crabs and many kinds of fowl. Ducks and other waterfowl hunting with snares took place in salty shallows during spring and autumn migration seasons, and some of the meat was preserved [24]. Wild boar, wolf, fox, weasel and hare were also hunted [23, 25, 34], as was the Caspian tiger, *Panthera tigris virgata*, now extinct [75]. The tiger was mostly hunted with poison or iron traps (*tozaq*) for fur which was traded for other goods [63]. Tiger flesh medicine was popular among the Chinese, and traders paid high prices for tigers [23, 34, 40, 50, 55]. A place was even called *Jolbarš äsildi* ‘Where the tiger was killed,’ probably an experience-based toponym [63].

Other fur and skin animals the Loptuq hunted were Eurasian otter, *Lutra lutra*, red foxes, *Vulpes vulpes* and whooper swans, *Cygnus cygnus*. All these were bartered with traders or peasants in settlements [49, 66]. Local *begs* also collected tax in furs, and the Loptuq paid yearly a certain amount of otter skin to the *amban* of Turpan. Wild Bactrian camels, *Camelus bactrianus ferus*, were killed at water spots in summer and autumn by specialized hunters, often in the Kumtagh desert, for their wool, which fetched high prices in towns like Turpan [76]. The Loptuq ate the meat and made footwear of the skins. Already in the 1870s, camels were decreasing in number and today they are almost extinct [22, 23]. In the summer, some Loptuq escaped from mosquitoes and other insects in the marshes to the mountains, where they hunted wild yak, *Bos grunniens mutus*, and Asian wild ass, *Equus hemionus*, for meat and hides. Hunters would also leave for the Tibetan plateau in autumn to obtain fur animals such as Tibetan antelopes *Pantholops hodgsonii*, Maral deer, *Cervus elaphus yarkandensis*, and wild horses, *Equus ferus przewalskii* [49]. Being Muslims, however, the Loptuq did not hunt wild boar, *Sus scrofa* L., 1758 [77]; therefore, according to some explorers, the numerous boars did not fear humans [23, 25]. Snares were set among the reed in order to catch ducks. Also swans were taken with snares [33].

**Fig. 8:**
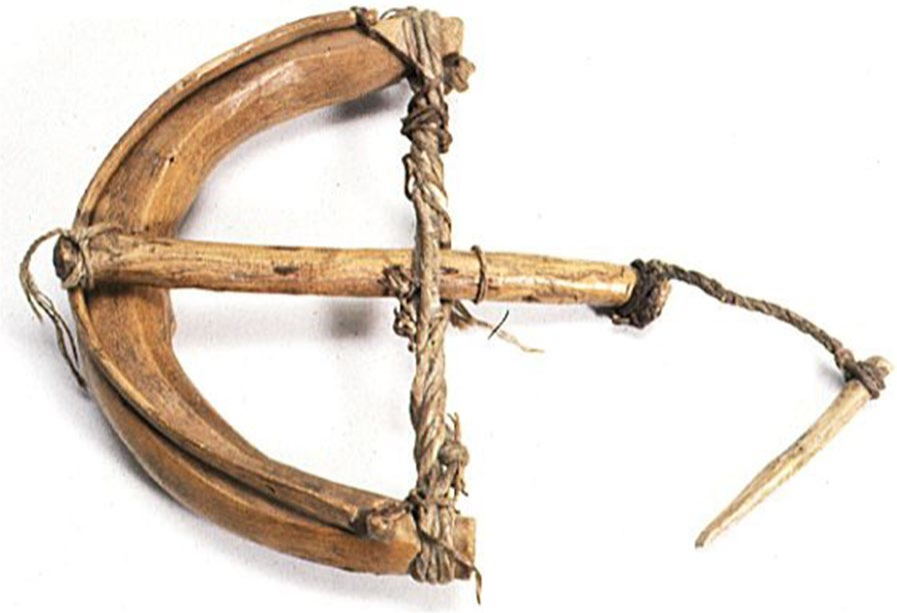
Torsion trap made of wood and fibres for capturing pigeons and chukars. Gathered by Nils Ambolt in Cherchen village during the Fourth Hedin expedition in the 1930s (Hedin-Bendix Collection, Museum of Ethnography, Stockholm)

Animal skins and bird feathers were utilized for clothing purposes. A traditional winter headdress was a sheepskin cap, and men could wear a fox fur hat in winter and a lighter felt cap in summer. In winter, women wore duck-feather hats with feathers facing to the outside, and in summer they covered their heads with a scarf made of Lop hemp fibres. Swans provided downs used in clothing [33]. Duck feathers were sold to visiting pedlars [23, 24] (Fig. 8).

Except daily garments the Loptuq also had festive clothing. At Qara-Qoshun at least, people habitually slept without taking off their clothes. They also thought that to avoid sudden fire or robbery, it was best to keep extra clothes far from home, and bury the clothes in the sand; only they knew where [23].

The Loptuq normally walked bare-footed, but in wintertime, they wore *čoruk,*simple shoes made of undressed hide. In winter, they usually lined their cloaks with duck-skins dressed with salt. When animal husbandry increased among the Loptuq at the end of the nineteenth century, sheepskin coats appeared. According to Huntington, the Loptuq summer dress in 1905 consisted of quilted common tunics reaching below the knee, high leather boots and fur-brimmed caps [23, 27].

### Plants for construction, canoes and fuel

**Fig. 9:**
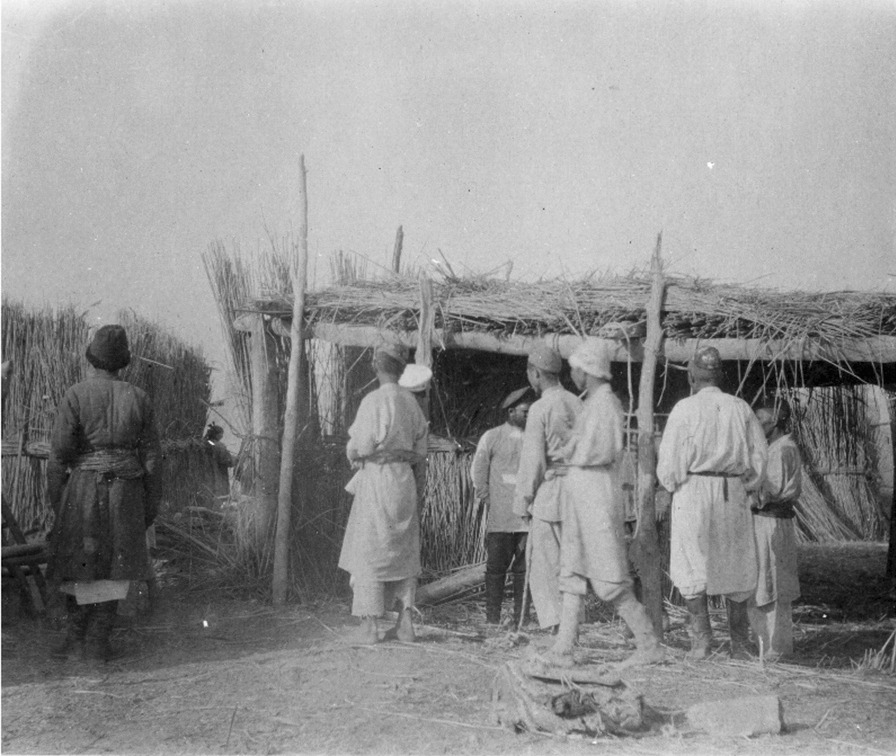
Loptuq men constructing a *satma,* a new reed hut with wooden structure at the hamlet Dilpar in 1934 (Photo David Hummel)

The common reed, *Phragmites australis*, in the Loptuq vernacular referred to as *qamïš* [31], was abundant and in addition to food also essential for the construction of huts, *satma* or *qamïš öj* (Fig. 9)*,* furniture and as fuel. In the autumn, reed panicles were gathered for making beds [23]. Reed provided many services also for the culture: corpses were placed on a stretcher made of reeds at funerals, and often also covered with reeds [33]. In the reed belts, there was a kind of grass known as *aqčï*, identified as *Arundinella nepalensis*, which was used for making baskets and mats [63] (Table 5).

**Fig. 10:**
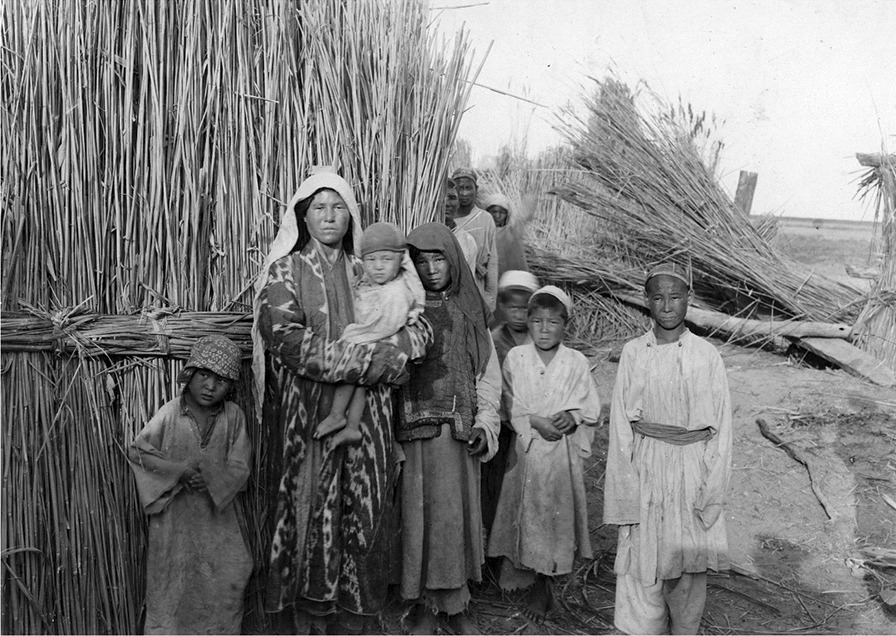
Loptuq household in front of a reed hut around 1900 (Photo Sven Hedin)

Huts were generally constructed with a framework of poplar, *Populus euphratica* logs. Corner logs, *tuluk,* roof beams *baraj*, and smaller joints, *čäsijaγač,* were important to keep the structure together. Vertical reed bundles were tied to the logs, and the earthen floor covered with reeds. A flat reed roof was added, with the hope that it would keep against the rough handling by the winds [78].Three categories have been identified by Uyghur ethnographer Häbibulla: wall house (*tam öj)* made of wood; adobe house (*čaplima öj)* made of reed, covered with a layer of straw and mud; and reed house (*qamïš öj)* [59]*.* Basement houses, *geme*, are huts dug out in the ground and built of branches and reed [55, 63, 70], while *alčuq* is a small wooden shed [63] (Fig. 10).

A house was never demolished if a Loptuq family had to move elsewhere, because they might return at a later point; and if someone else chose to settle in the area, they could use the house [67]. Henri d’Orléans noticed swallow nests inside the huts and was told that birds were greatly respect [33].

**Fig. 11:**
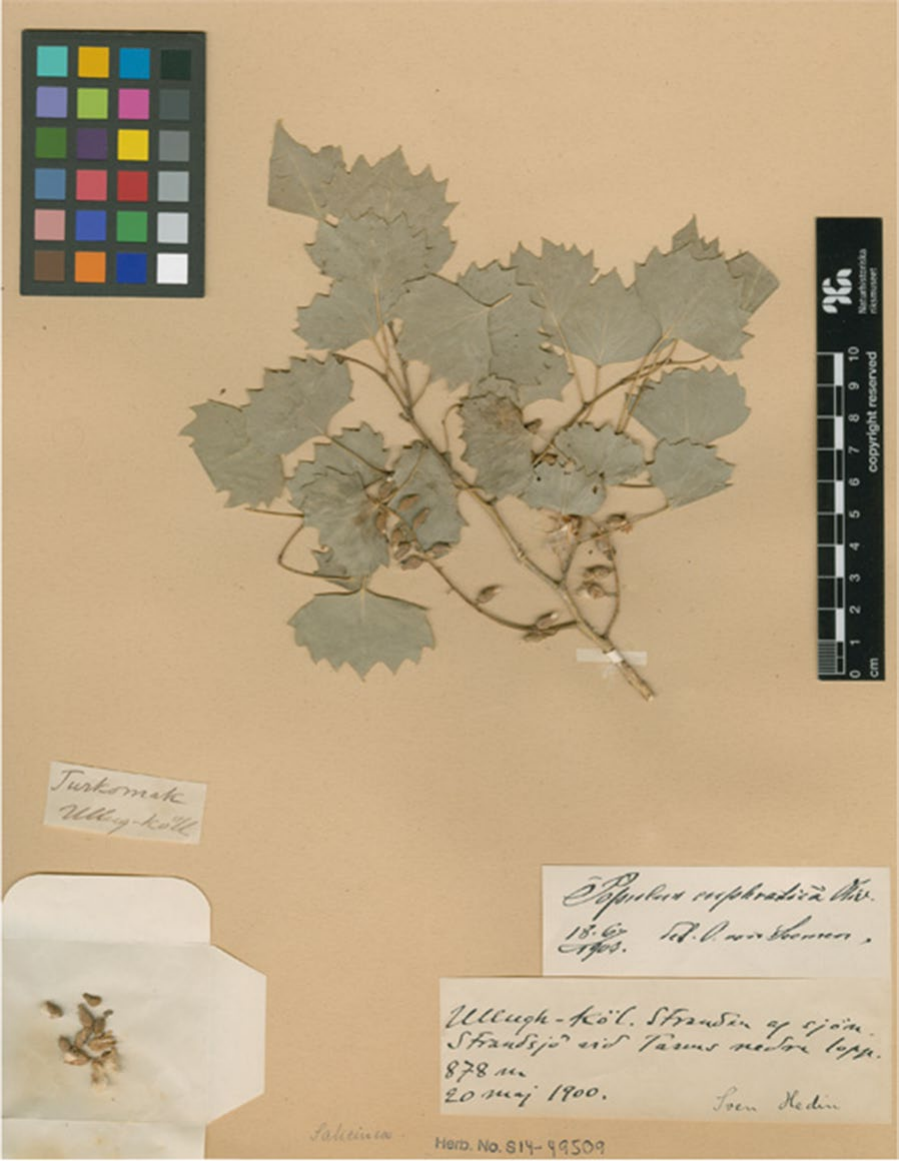
Voucher specimen of the common poplar tree in the Lop Nor region, *Populus euphratica*, gathered by Sven Hedin at Ulugh Köl, 20 May 1900 (Swedish Museum of Natural History, Stockholm)

**Fig. 12:**
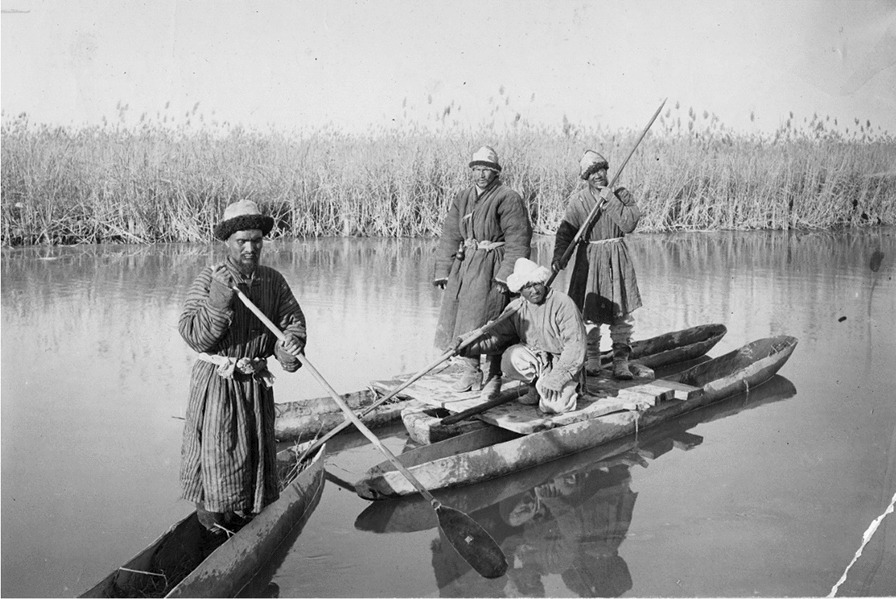
Loptuq fishermen in their dugout canoes made of poplar; two or more canoes would be tied together for more stability and space while transporting or fishing (Photo Sven Hedin ca. 1900)

Euphrates poplar, *Populus euphratica*, locally known as *toγraq,* was used for dugout canoes (*kemi*) (Figs. 11, 12). The poplars must grow at a distance from the water to be of good quality for a canoe [21, 31, 43, 55]. Besides being used for water-borne transport, the dugout canoes served as symbolic items during weddings and funerals. According to a recent Uyghur source, it was customary among the Loptuq to provide the bride with a bride-wealth (*qalïŋ*) [31, 33, 79], which consisted of seven or nine fish, seven or nine pieces of deer skin, a pair of dugout canoes and a fishing net, a couple of hooks, seven or nine pieces of cloth made of *Poacynum*-fibres, and a few bags of *jigde,* fruits of *Elaeagnus angustifolia *[79]. Such wedding gifts were also mentioned by travellers to the region. According to Przhevalsky, the bride-wealth consisted of ten bundles of Lop hemp fibre, ten strings of dried fish, and one hundred to two hundred of ducks [24]. After the 1930s, however, these customs changed to also include products such as cow and sheep skins, different types of clothing and wheat flour [79]

Five categories of poplar canoes were documented: *beliqči kemi* (*kölče kemi*) ‘fishing canoe’, a small, maximum two-person light vessel for transport and fishing; *šal kemi,* a wide wooden trunk canoe; *čong kemi* ‘big boat’ which could carry as much as 500 kg, mainly used for transporting people and livestock; *sal kemi* (*qoš kemi, četiq kemi*), a cluster of two, three or four canoes, capable of carrying a cargo weighing a ton; *leylime kemi,* a dozen of vessels tied together with a tree trunk laid over, carrying up to two tons of cargo. Hedin saw canoes up to eight metres long and almost a metre across. He noted that the Loptuq lived “half their lives” in their canoes, and were very apt at moving fast and quietly or noisily if needed while fishing. A good canoe lasted for eight to ten years; it took around five days for three men to make a new canoe out of a fresh poplar trunk. On the open lake, the rowers would kneel, but in the reed belts they stood up to see better and to punt the canoe. Two people often rowed or punted together, the man behind standing up and the one in front kneeling [25, 40, 61]. According to Przhevalsky, dugout canoes were used as coffins and the deceased was buried together with half of his fishing-nets [23, 24]. For land-transports the Loptuq used wooden carts such as *jarijar araba, tömür araba* and *čirga,* depending on the need for labour and transport [79, 80].

Tamarisks, *Tamarix androssowii*, *Myricaria davurica* and other species of the family Tamaricaceae had several uses, including fuel. Several tamarisk species occurred in the vicinity of the Loptuq settlements. According to Malov, *puta* is the word for tamarisk in Loptuq, and a common component in many toponyms; a generic name *julyun* was also used [31, 65].*Myrica davurica*was locally known as *malγun* or *balγun*, which also occurs in toponyms such as Bash Balghun and Qash Balghun [63]. Hedin found a graveyard between sand dunes and scattered bushes of tamarisk a few hundred metres away from a village. Each grave was marked by a high pole with a horsetail, and a fence of horizontal tamarisk branches surrounded the graveyard [25]. Gathering forage was an important source for cash for the Loptuq. They sold the gathered forage to passing travellers, but they did not gather fodder for themselves, as long as they could subsist mainly on fishing [35].

**Fig. 13:**
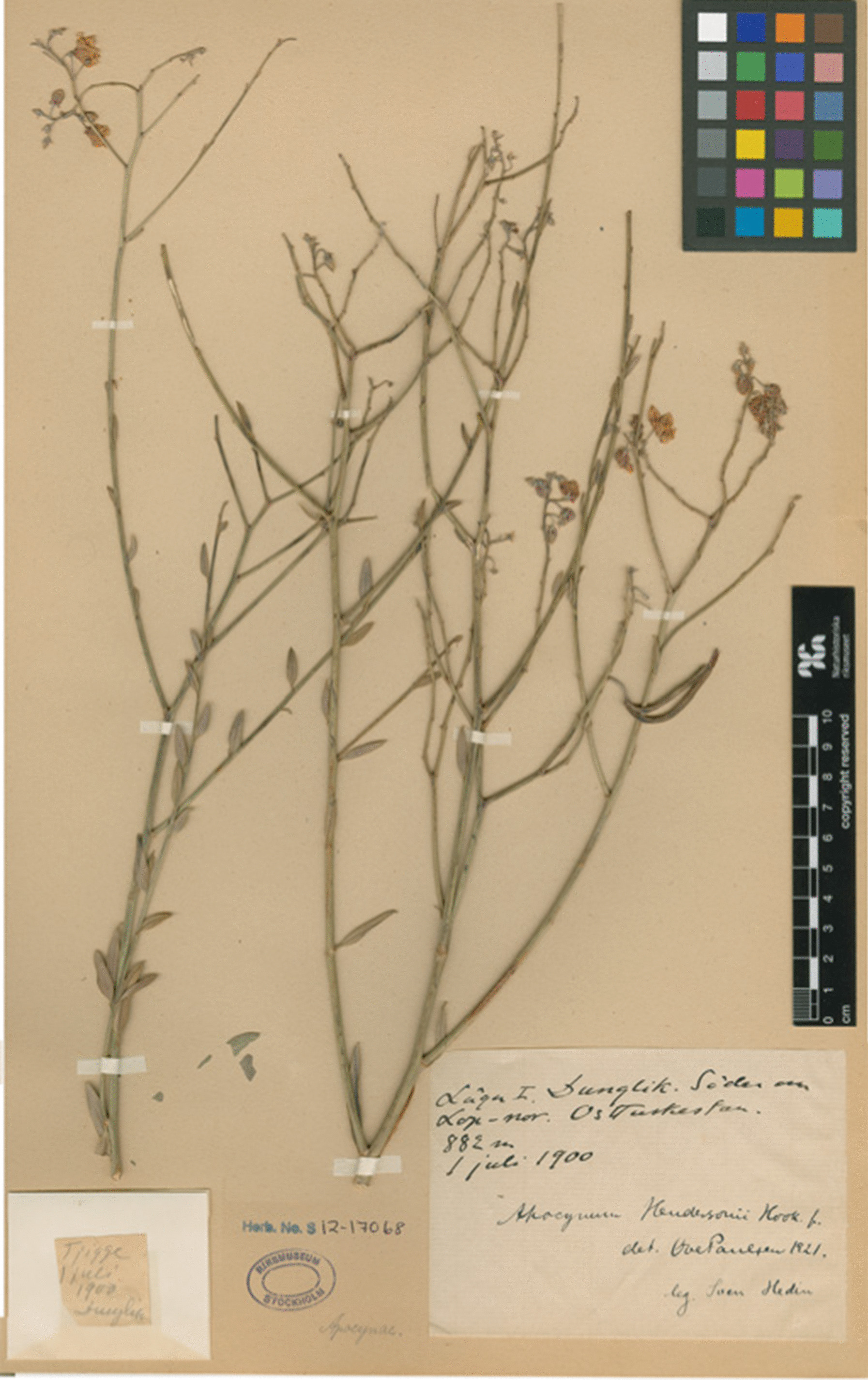
Voucher specimen of Lop hemp, *Poaocynum pictum*, gathered at Dunglik south of Lop Nor, by Sven Hedin on 1 July 1900 (Swedish Museum of Natural History, Stockholm)

Many sources describe how essential *čige, čigä* or Lop hemp, *Poacynum pictum* (*čige* probably also includes *Apocynum venetum* L.), was for the Loptuq (Fig. 13). This hemp grew abundantly in the vicinity of their settlements and was easy to harvest [81]. Many items were made from this product, including fishing nets, strings, sails and garments. The explorers described the fibres as being strong and of high quality [23, 25, 33]. According to Huntington, the fibres obtained from the inner bark of the Lop hemp were much tougher than ordinary hemp and stronger than cotton [27].

**Fig. 14:**
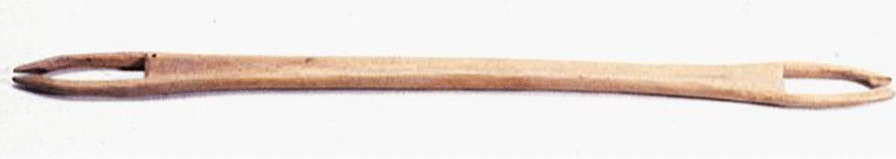
Wooden needle for knitting fishnets. Collected by Georg Söderbom in Charchak at Kum-darya during the Hedin expedition in May 1934 (Hedin-Bendix Collection, Museum of Ethnography, Stockholm)

**Fig. 15:**
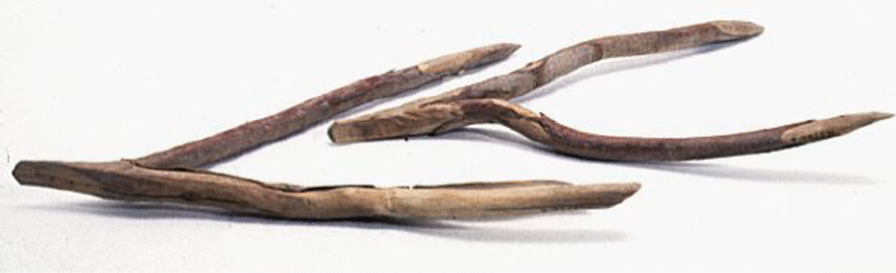
Wooden loom frame. Consists of two branch “feet” on which a crossbar is attached, and a board with wide holes in which the two branch feet are inserted, and a weaving sword. Collected by Georg Söderbom in Charchak at Kum-darya during the Hedin expedition in May 1934 (Museum of Ethnography, Stockholm)

Prince Henri d’Orléans noted in 1889 that the plant was first uprooted with a hoe made of a triangular piece or iron; a piece of reed served as the handle. The stems were cut with a hatchet, made of a fragment of iron and a piece of bent wood [33]. For fishing nets and other products, the leaves of the Lop hemp were first dried and then boiled in water for half a day [27]. Jarring, quoting Hedin’s unpublished diary from the first visit, notes that when the fibres “have turned soft, they are worked into fine soft threads, which are twisted into strings, which in turn are twisted two together into strings, and tied into nets with diagonal meshes” [63] (Figs. 14, 15)

**Fig. 16:**
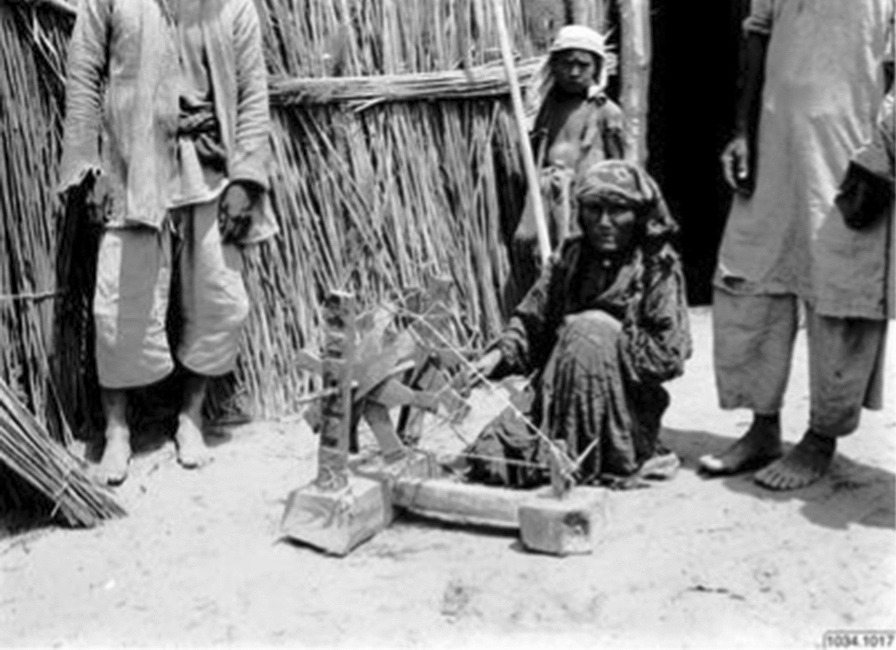
Old Loptuq woman showing a simple spinning wheel (Photo Sven Hedin)

In addition to fishing nets, Lop hemp fibres were essential for making cloaks and trousers. The weaving of garments was always done by the women. Katanov provided a rather detailed description of how the *čigä* fibres were prepared and made into cloth by Loptuq women: they gathered plants in the shrubby lands and then soaked the harvest in water. After a few days, they would take out the plants and peel off the bark; after five or six days, the hemp would begin to putrefy, stink and eventually separate into individual fibres [74]. According to Hedin’s unpublished diary from his second expedition to Eastern Turkestan in 1899–1902, he observed an old woman “sitting there, beating *chigge*-fibres until they became fine and white as cotton, ready to be used for making sack cloth, *tagar*” [63] (Fig. 16)

*Poacynum*-fibres was also used for the production of a number household items such as soft beds for children. According to Bonvalot, cushions “as soft to touch as the finest velvet” were manufactured from the silky substance around the grain [33].

**Table 5:** Other utility plants

Scientific name	Use
*Poacynum pictum* (Schrenk) Baill.	Fibres for clothes and fishnets, medicine, bride- wealth
*Arundinella nepalensis* Trin.	Baskets, mats
*Populus euphratica* Olivier	Construction material, utensils, tools
*Phragmites australis* (Cav.) Trin ex. Steud.	Construction material, fuel, beds
*Myricaria germanica* (L.) Desv.	Fuel
*Myricaria davurica* (Willd.) Ehrenb.	Fuel
*Tamarix androssowii* Litv.	Fuel
*Alhagi kirghisorum* Schrenk	Forage, fuel
*Lactuca tatarica* (L.) C.A. Mey.	Forage
*Krascheninnikovia ceratoides* (L.) Gueldenst.	Forage, fuel
*Ephedra przewalskii* Stapf	Folk medicine

### Household goods

**Fig. 17:**
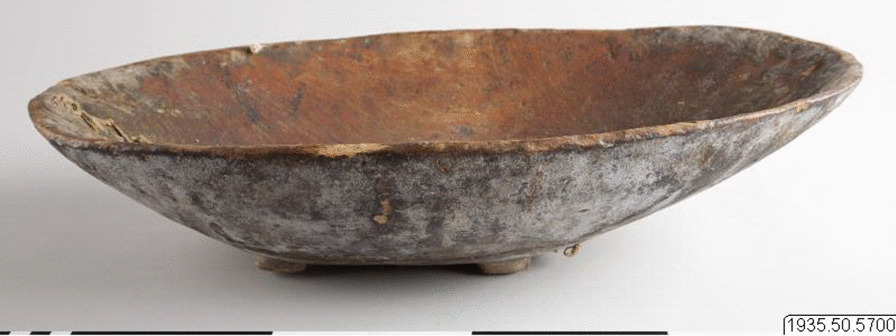
Trough made of poplar wood used in a Loptuq fisherman’s household. Collected by Georg Söderbom in Charchak at Kum-darya during the Hedin expedition in May 1934 (Hedin-Bendix Collection, Museum of Ethnography, Stockholm)

**Fig. 18:**
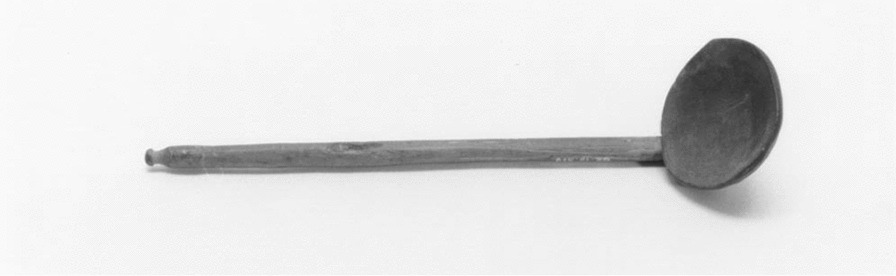
Wooden ladle collected by Sven Hedin in 1902 (Museum of Ethnography, Stockholm)

Loptuq families kept only a small number of tools and household utensils in their homes. Most were made of poplar wood, but a few iron items were manufactured in the nearby towns Charkliq or Korla and bought from peddlers who yearly visited the settlements. Sven Hedin made a list of household items he found with a family in a small village: three old and three new canoes, a few poplar trunk buckets (*soγul*), a big cauldron (*qasan*) for cooking fish, some iron jugs (*čugun*), wooden bowls (*ajaq*), canoe hand bailers, big willow baskets for storing wool and Lop hemp fibre, spinning wheels (*čarq*) [82], tools for twining ropes (*čarq-iγi*), axes (*kejči*), grindstones (*billy*), knives (*bičaq*) of different sizes, scissors (*qaiči)*, awls (*derepči*) for making holes for string in skins, snares (*suγan*) to catch duck, and oars (*gädʒäq*) [25, 31, 40, 63] (Figs. 17, 18).

**Fig. 19:**
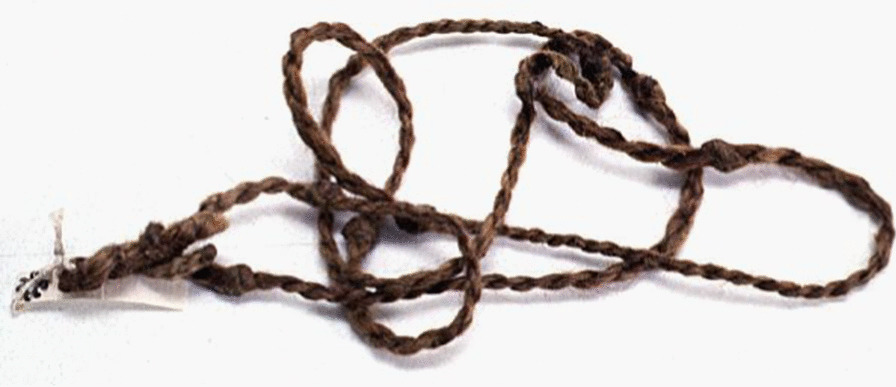
Loptuq protective amulet made of camel wool yarn. “The evil spirits must be afraid of camels. The mullah priest [prayer leader] tied eight knots on the string, then gave it to the boy to wear around his neck”, Georg Söderbom noted. Collected by Georg Söderbom in Charchak at Kum-darya during the Hedin expedition in May 1934 (Hedin-Bendix Collection, Museum of Ethnography, Stockholm)

Russian travellers noted that the women had a limited number of items at their disposal. Besides clothes, these would consist in spindles, knitters, at times simple weaving frames, a few needles and other small items. Almost all the men had leather-sheathed lighters, razors and knives that they carried in their belts. The men also carried wooden or leather snuff boxes in their shirts; they chewed the tobacco. Oars, nets and fishing rods, dried fish ties and hemp were shared by families; common property included traps used for hunting foxes and wolves. Only a few Loptuq men owned guns [23, 34] (Fig. 19).

### Ethnomedicine

As mentioned above, tigers, *Panthera tigris*, were killed with traps or poisoned by the Loptuq for medicinal purposes of the Chinese. Knowledge about traditional remedies used by the Loptuq themselves is, however, limited. Hedin noted that the flesh of the *köl buqa* ‘lake bull’, “on account of its curious cry”, or Eurasian bittern, *Botaurus stellaris* (L., 1758) was used as a remedy against tuberculosis [55]. Blood of shrike, *Lanius sp*., was used to promote pregnancy and as a diuretic [55]. According to Forsyth, the Loptuq referred to smallpox as *jaman* ‘the evil’ disease, and they adhered to a kind of self-imposed quarantine lasting forty days [22].

An interesting shrub with stubby stems, roots and branches is the *čakanda* or *čakande*, probably *Ephedra przewalskii,* which grew abundantly in the region [83, 84]. Folke Bergman, who accompanied Hedin on his last visit, mentions that in addition to its medicinal value, its often brilliant features caused it to be considered a symbol of life by the Loptuq [85]. Interestingly, although there is no evidence of any connection between historical Loulan near Lop Nor and the much later Loptuq in the area, almost every known grave of the Loulan or Qäwrighul culture (2000–1700 BCE) contains carefully bundled twigs identified as *Ephedra* [86, 87]. According to Tarim mummies expert Victor H. Mair, the deceased were accompanied by a small bag of *Ephedra*, which was probably employed for medico-religious use [88]. This would indicate that *Ephedra* has been present in the area for the past four millennia.

Reflecting their fishing and foraging culture, it is perhaps not surprising to observe that among the Loptuq, fish bones were commonly used in divination for predicting the future through magic ritual [69]. According to Häbibulla, fortune-telling through bone reading was performed by throwing the backbones of a fish into the fire. The size and thickness of the appearing blisters, and the width of the spaces between the blisters, were then interpreted [70].

The Loptuq used a kind of wild yeast, *toγräγu,* as a remedy against snake-bites, but also as leavening agent in baking. It was a substance derived from the trunk of *Populus euphratica,* but if it was a fungoid growth or a sort of gum, we cannot ascertain. The Loptuq differed between two kinds, *aq* ‘white’ and *qara* ‘black’ *toγräγu* [22]. According to Forsyth, snake bites were frequent in the Lop Nor area. There was a snake called *dúr* whose bite was fatal and very much dreaded [22]. We have not been able to establish the scientific name of *dur*; it may be a type of venomous pit-viper [90].

## Discussion

Specialized fisher-forager communities can reveal much valuable information about human adaptation to difficult ecological conditions and harsh climates, but they have been little studied so far. Moreover, the changes, often triggered through contact with the outside world, are important to research. In this article, we have demonstrated that the material culture of the Loptuq was shaped not only by physical but also cultural imperatives: the physical environment put constraints on what was possible, and yet they used the ecological situation to create a unique culture in the heart of the desert [91]. However, this culture was destroyed by the Chinese authorities when they forcibly resettled the Loptuq far away from the Lop Nor area in the 1950s. The Lop Nor desiccated site was then used for nuclear bomb testing until 1996 and is now utilized for mining potash. Today the Loptuq are assimilated with the Uyghur, and the only sources about their language and culture which remain are those by visitors to the area around 1900 [25, 61].

The Loptuq are today largely ignored, censored or simply deleted from history in P.R. China. They are for political reasons seldom, if at all, mentioned in modern sources about the Lop Nor area; the displacement of this people and destruction of their habitat is even being denied [61]. Their experience and knowledge, however, could be useful today for other groups, living in botanically scarce habitats or at the fringe of deserts, in the present challenging period of climate change when deserts are expanding at a staggering rate. It is also imperative that researchers try to preserve and reconstruct extinct cultures, as they contribute to the global knowledge about human life on this planet.

**Fig. 20:**
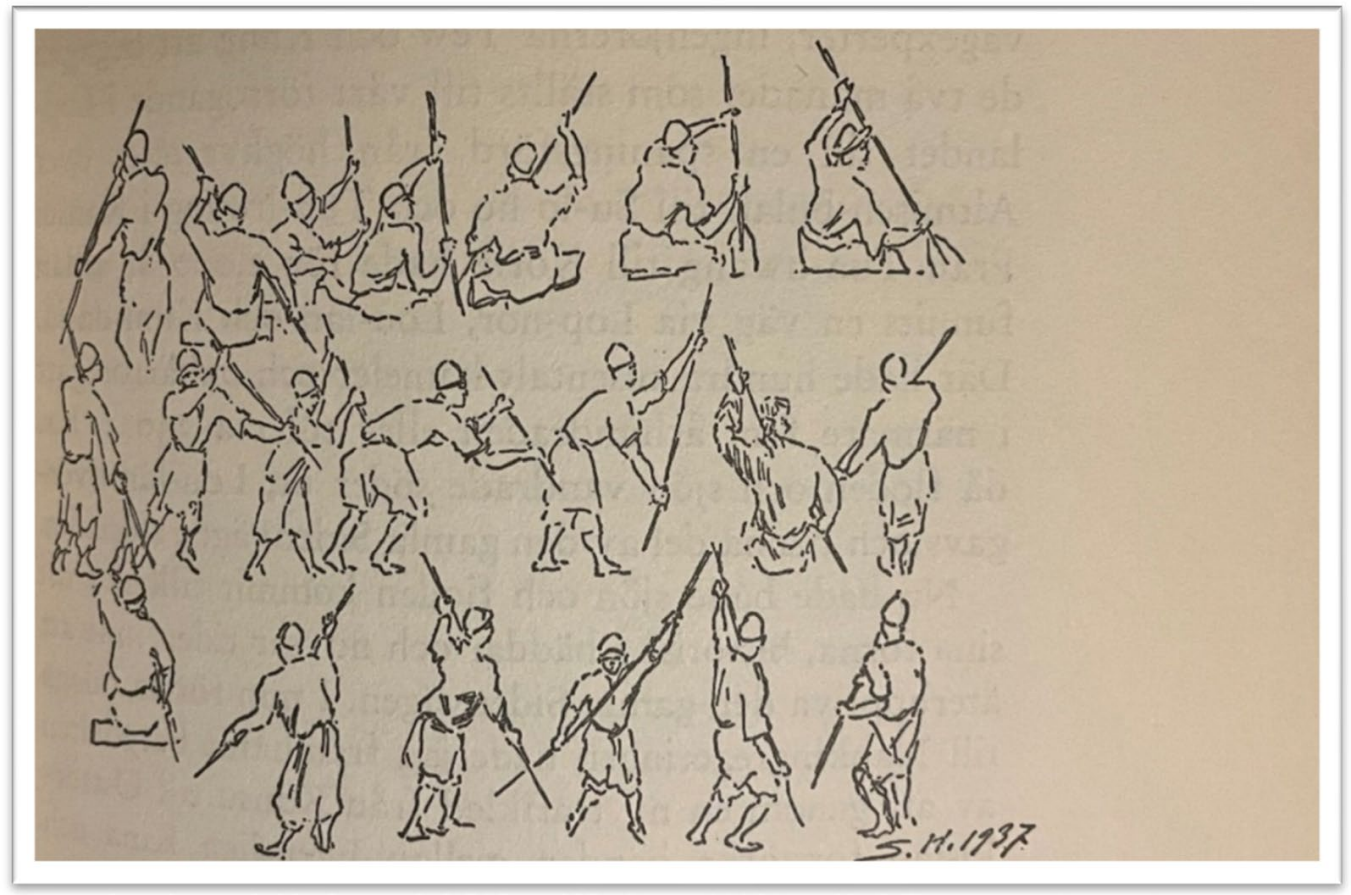
Loptuq techniques for propelling forward a dugout canoe (Drawing by Sven Hedin 1937)

The Loptuq made the most of scarce biological resources in the surrounding desert, lake and marsh environment. Regarding botanical resources, three plant species dominated the raw materials utilized for buildings, textiles and tools around the end of the nineteenth century, when Sven Hedin and other travellers visited the Loptuq. These three were *Phragmites communis*, *Populus euphratica* and *Poacynum pictum*. Still in the 1930s, when Hedin again visited the Loptuq, these species continued to be important for their everyday life (Fig. 20). Like most subsistence economies [92], the Loptuq knew, used and keenly observed the local plants, animals and other organisms, and followed their growth and availability. They were also knowledgeable about other plants in their neighbourhood, including taxa which they named, but which were of no use to them. Besides the three obvious species used in their material culture, some others were used for food, medicine and similar purposes. There could of course be further plants, which were not recorded by the visitors of over a century ago, although Hedin who remained with them for over a year was very thorough.

Globally, it is known that peoples living in desert environments have possessed a vast knowledge about wild plants; for instance, the Tuareg nomads in the Ahaggar Mountains in central Sahara had names for around 500 plant species. They knew which ones could be used as feed for camels, goats and sheep, and which ones were for ethnoveterinary purposes or usable as supplement for their own food [93]. The !Kung San, who lived as hunter-gatherers in the Kalahari desert, knew between 400 and 500 species, and at least a hundred of them were used as food for humans [94]. For the Haddad and other small ethnic foraging groups living around Lake Chad, plants were important for nutritional needs, although the lake also provided fish, mammals, crocodiles and other game [8].

These examples from Africa, however, are from areas with much richer biodiversity than the Taklamakan Desert and Lop Nor area. Both the flora and the fauna of the Lop Nor region were poor and have become even poorer today. Another reason for the limited species used by the Loptuq could be that they were fairly newcomers in the area, having only had a couple of hundred years to adapt to the region; several travellers heard stories about migration some 300–400 years ago [21].

Studies of ecosystems with reed vegetation and marsh landscape indicate that nutrient availability is very high. Reed belts can support 9.4 human beings per square kilometres. Fish and seafowls are readily available food. In addition, rootstocks of reeds also have good nutritional value [95]. The Loptuq environment was no exception to this, as reeds and other aquatic plants provided a variety of services for the fisher-foragers living there.

It should also be noted that the Loptuq did not live as isolated as several of the explorers wanted to make their readers believe. Certainly, the paths through the desert to the Lop Nor were perilous and difficult, and required desert skills and much persistence, but there were itinerant peddlers and traders, who visited yearly and both sold goods and bought products such as tiger meat, furs and skins from the Loptuq. Historical sources can, as Kevin Kind has recently shown, teach us about epidemics that also affected the Loptuq, such as a smallpox epidemic of second half of the eighteenth century that was fatal to their survival [96].

Around 1900 some Loptuq had started to move away to small towns in the vicinity, finding jobs or migrating for seasonal work. The men also went on long hunting excursions to other regions, and whole families moved to the mountains when mosquitoes became too many in summer. Thus, the Loptuq were already in a process of transforming their subsistence and livelihood.

Artisanal fishing cultures are disappearing globally and so is local knowledge of the fish and other aquatic biota they depended on for their subsistence. Historical ethnobiology can help us save knowledge of these cultures, which have been very important throughout history not only locally, but also for the global diversity of cultures and human life [97].

## Conclusion

Over several generations, the Loptuq developed adaptation strategies to the specific conditions and aridity of their environment, as well as ways of fulfilling their material needs. They made the most of locally available resources, which enabled their survival in this area, which was so poor in species.

This study shows how historical source materials can be analysed from different viewpoints and approaches to extract much interesting data for ethnobiologists, ethnographers and linguists. Combining multiple sources such as travel reports, recordings of toponyms and other linguistic data, and material culture objects in museum collections, drawings and photographs, makes it possible to gather and even reconstruct a specific group’s relationship with the environment and its biota, and how biodiversity has enabled the group to survive even in arid, and for many uninhabitable environments. This is the case with the Loptuq: most of the explorers who documented them had difficulties understanding how they could live in such a place.

It is also important to contextualize folk biological data as much as possible, to understand the human–biota relationship. The Loptuq fishing-gathering culture reflects an adaptation to a specific ecosystem in the marshlands of the Lop Nor lake, and along the shores of Lower Tarim and Konche Rivers. The Loptuq knew and named several plants and animals in their habitat, and also employed phytonyms and zoonyms for toponyms. There are, however, several biocultural domains for which we have hardly any information, such as ethnoveterinary and folk medical uses. In a comparative perspective, the three main plants they used, reed, poplar and Lop hemp, and the few others they harvested, comprise a very small number of plants, whereas in other places with richer biodiversity, the examples of plant and animal use is naturally larger. These few plants were, however, used for a multitude of purposes by the Loptuq, satisfying their everyday needs. They took care of the environment, changing it for easier transport and fishing (channels through the reeds), and through close observation and naming the growth pattern of plants, occurrence of animals, etc.

It is important to emphasize that their lifestyle, subsistence pattern and local culture were in no way static but in constant transformation, due to ecological, political, social and economic factors. Part of these they could manage, the ecological changes in their immediate environment, but political, social and economic influences from outside their settlements intruded increasingly at the end of the nineteenth century and eventually changed their way of life radically in the 1950s, when they were displaced and forcibly assimilated by the Chinese authorities with the larger Uyghur population. The Loptuq had trade contacts before the foreign visitors appeared, and they had started small-scale farming and some kept sheep. The narrative of the lonely Loptuq fisher-gatherers, waiting to be discovered, is a story created and disseminated by visitors from Europe and America, whose main interests were exploration (and subsequent fame, and funding for their next journey). Only Sven Hedin remained with the Loptuq for a longer period, learning and observing their everyday life.

Historical ethnobiology deals with human lifestyles and relationships with the biota and land- and waterscapes which have disappeared. Our knowledge of living conditions, habits, cultural expressions and local familiarity with the land and waterscape surrounding the Loptuq is bound to remain limited due to the destruction of their culture. Naturally, when their ecosystem and lifestyle changed, their relationship with the biota had to change. We face a source problem: these only exist over a span of some fifty years, and few tell more than basic facts. Therefore, comparing them and using source pluralism, we must “read between the lines” and combine information in diverse ways to extract the most we can, just as the Loptuq extracted whatever they needed and could from reed, poplar and Lop hemp. Most of the knowledge about their environment and biota will remain unrecorded, although linguistic documentation can still be used to discover further knowledge. Their former lifestyle, which was closely associated with the ecological conditions in the swamps and waters of Lop Nor is now gone, but the memory of their cultural heritage can and should be studied using the preserved documents and artefacts.

## Data Availability

The data supporting the conclusions of this article are included within the article. The collection of Loptuq tools is kept in the Museum of Ethnography in Stockholm and in the collection of the Sven Hedin Foundation at the Swedish National Archives. Voucher specimens of plants from Lop Nor and Lower Tarim area can be found in the herbarium of the Swedish Museum of Natural History in Stockholm.

## Abbreviation

P.R.: People’s Republic
